# Unveiling the prevalence and antimicrobial resistance landscape of *Pantoea* genus in veterinary clinical strains: Insights from a cohort study

**DOI:** 10.1016/j.onehlt.2025.101130

**Published:** 2025-07-07

**Authors:** Ondrej Holy, Vladimir Sladecek, Jaroslav Bzdil, Monika Zouharova, Julio Parra-Flores, Cátia Caneiras

**Affiliations:** a Science and Research Centre, Faculty of Health Sciences, Palacky University Olomouc, Hnevotinska 3, Olomouc 775 15, Czech Republic; b Ptacy s.r.o., Valasska Bystrice 194, 756 27 Valasska Bystrice, Czech Republic; c Veterinary Research Institute Brno, Department of Infectious Diseases and Preventive Medicine, Hudcova 296/70, Brno 621 00, Czech Republic; d Department of Nutrition and Public Health, Faculty of Health and Food Sciences, University of Bío-Bío, Chile; e Environmental Health Microbiology Laboratory, TERRA Associate Laboratory, Institute of Environmental Health (ISAMB), Faculty of Medicine, University of Lisbon, Lisbon, Portugal; f Egas Moniz Centre for Interdisciplinary Research (CiiEM), Egas Moniz School of Health and Science, Monte da Caparica, Portugal; g Institute of Preventive Medicine and Public Health, Faculty of Medicine, University of Lisbon, Lisbon, Portugal

**Keywords:** Animal, Environment, *Pantoea agglomerans*, One health, Public health, Antimicrobial resistance

## Abstract

The genus *Pantoea*, of which *Pantoea agglomerans* is the most common species, is an emerging Gram-negative facultative anaerobic bacillus that causes a wide range of opportunistic infections. To date, the prevalence, antibiotic resistance, and pathogenic potential of this bacterium in animals remains largely unexplored. The aim of this work was to describe the prevalence of microorganisms of the genus *Pantoea* in clinical samples obtained from animals during the period 2015–2017 and to define their susceptibility to antimicrobial agents. In the monitored period, a total of 23,739 clinical samples obtained from animals in the Czech Republic with symptoms of disease were tested, from which 151 *Pantoea* genus were isolated (prevalence 0.63 %). Cultivation and incubation were carried out under aerobic conditions by culture methods using massopeptone blood agar, Endo's agar and xylose lysine deoxycholate agar at 37 ± 1 °C for 24 h. Suspect strains were confirmed by matrix-assisted laser desorption/ionization coupled to time-of-flight mass spectrometry (MALDI-TOF MS). Susceptibility testing was performed by the standard disk diffusion method using Mueller-Hinton agar. *Pantoea* strains were recovered from domestic horses, carnivores (dogs, cats) and rodents (prevalence of 6.78, 1.64 and 1.12 % respectively). Resistance to beta-lactam antimicrobials was detected in 12 strains. In addition to beta-lactams, resistance to co-trimoxazole was detected in 1 case and to co-trimoxazole and chloramphenicol in 1 case, highlights the need to monitor the emergence of this strain in the context of the One Health approach.

## 1 Introduction

In recent years, even in veterinary practice, there has been an increasing recognition of microorganisms previously considered saprophytic, now isolated from pathological processes and lesions in animals. Among them are species from the genus *Pantoea*, part of the *Enterobacteriaceae* family. This genus includes diverse species such as *Pantoea agglomerans* and *Pantoea ananatis*, commonly found in a wide range of ecological environments [1].

The genus *Pantoea* displays remarkable ecological versatility. *Pantoea* strains can act as plant symbionts, promoting growth through nitrogen fixation in their leaves and roots, phytohormone production, and disease suppression [[1], [2], [3]]. In particular, *P. agglomerans* produces many antimicrobial compounds, such as herbicolin, pantocins, microcin, agglomerins, andrimid and phenazine, which are active against both Gram-positive and Gram-negative microorganisms [3]. In addition, some *Pantoea* species contribute to environmental bioremediation of soil, water and sediments through their ability to degrade contaminants (e.g., phenol, mesotrione, hydrocarbons) and absorb heavy metals such as copper, chromium, cadmium and arsenic [1].

In insects, including bees, *Pantoea* strains are symbionts found in the ovaries and digestive tract of each individual and can have a positive effect on the process of metamorphosis and play an important role in their defence against insect pathogens and predators as defensive symbionts [4,5]. These bacteria help in nutrient synthesis, detoxification of plant defences, and digestion of lignocellulosic plant matter [6,7]. Lipopolysaccharide IP-PA1, which activates macrophages, has a wide spectrum of effects and can be used in the prevention and treatment of cancer, hyperlipidemia, diabetes, ulcers, various infectious diseases, atopic allergies and stress-induced immunosuppression in humans and animals. In addition, IP-PA1 has an analgesic effect [8] and can even inhibit morphine dependence [9]. Conversely, *Pantoea* genus also includes pathogenic strains. Several species such as *P. vagans*, *P. eucalypti*, *P. deleyi*, *P. anthophila*, *P. agglomerans*, *P. ananatis*, and *P. allii* can cause plant diseases such as blight in eucalyptus, brown stalk rot in corn, and postharvest onion rots [10,11].

Human infections have also been documented in the literature. Neonatal septicaemia caused by *P. agglomerans* and sepsis caused by *P. dispersa* have been described, some of which were fatal [12,13]. Other authors recovered 53 strains of *P. agglomerans* from human patients, of which 23 were from blood cultures, 14 from abscesses, 10 from bones and joints, 4 from the urinary tract, 1 from the peritoneum, and 1 from the chest. Findings were often associated with penetrating trauma associated with plant material and catheter-related bacteraemia [14]. Other authors described 14 findings of *P. agglomerans* from human patients, of which in 5 cases from wound infections, in 3 cases from urinary infections, in 3 cases from pneumonia and 1 case each from furuncle, bacteraemia and catheter infection [15]. Human infections of the endocardium, muscles, eye tissues, as well as nosocomial infections have also been described [16].

In animals, reports are limited but increasing. *P. agglomerans* has been isolated from equine placentitis [17] and hemorrhagic disease in dolphin fish (*Coryphaena hippurus*) [18]. In 2021, virulence factors such as the type VI secretion system, hemolysin, filamentous hemagglutinin as well as genes involved in iron uptake and sequestration were revealed by whole-genome sequencing in the *P. agglomerans* KM1 strain. Moreover, genes involved in the adaptation of the pathogen to environmental stress were also identified, which included factors ensuring resistance to osmotic lysis, oxidative stress, as well as heat and cold shock [19].

Antimicrobial resistance in *P. agglomerans* is variable. Some studies report resistance of up to 7.5 % to cephalosporins or semisynthetic penicillins in paediatric patients [14,20], while others document multidrug-resistant strains recovered from paediatric infections, with resistance to penicillins, cephalosporins, aminoglycosides, fluoroquinolones, and carbapenems [15]. Whole-genome sequencing analyses have identified resistance genes encoding resistance to antibiotics such as penicillin G, bacitracin, rifampicin, vancomycin and fosfomycin [19].

Given its complex profile—ranging from beneficial symbiont to opportunistic pathogen—the genus Pantoea underscores the significance for public health and highlights the relevance of the One Health concept, which recognises the interconnectedness of human, animal, and environmental health. Understanding the dual role of Pantoea species as both beneficial microbes and emerging pathogens is essential, as it underscores the need for vigilant monitoring, appropriate antimicrobial stewardship, and integrative, multidisciplinary strategies to address public health challenges effectively [21].

This work describes the occurrence of microorganisms of the genus *Pantoea* in clinical samples obtained from pathological processes and lesions in various organs and organ systems of animals in the period 2015–2017, and at the same time shows their sensitivity to commonly used antimicrobial substances.

## 2 Material and methods

A total of 23,739 samples of clinical veterinary material were examined on the territory of the Czech Republic during the observation period 2015–2017. All samples were collected from the skin, respiratory system, genitourinary system, digestive system, mammary gland, musculo-skeletal system, nervous system, eyes and bee brood by trained veterinarians during routine examinations of animals with disease symptoms according to standard sampling protocols. The exact number of samples to be examined is given in Table 1, Table 2.

**Table 1:** Prevalence of *Pantoea agglomerans* in the observed groups of animals and organ systems in the period 2015–2017.

Animal	Organ system	Number of examined samples	Number of positive samples	Prevalence (%)	95 % Confidence Intervals Wilson (%)
Carnivores (dog, cat)	Skin	1853	54	2.91	2.23–3.77
	Respiratory	618	11	1.78	1.00–3.17
	Urogenital	377	0	0	0.00–0.98
	Digestive	1436	3	0.21	0.07–0.65
	Eye	337	8	2.37	1.20–4.61
	Musculoskeletal	22	0	0	0.00–14.87
	**Total**	**4643**	**76**	**1.64**	**1.31–2.04**
Ruminants (cattle, goat)	Skin	31	2	6.45	1.79–20.55
	Respiratory	355	19	5.35	3.45–8.20
	Urogenital	128	0	0	0.00–2.85
	Digestive	632	1	0.16	0.03–0.88
	Eye	157	0	0	0.00–2.34
	Mammary gland	8822	6	0.07	0.03–0.15
	Musculoskeletal	3	0	0	0.00–56.20
	**Total**	**10,128**	**28**	**0.28**	**0.19–0.40**
Solipeds (horse)	Skin	92	5	5.43	2.34–12.18
	Respiratory	96	11	11.46	6.49–19.41
	Urogenital	9	1	11.11	1.98–43.47
	Digestive	48	0	0	0.00–7.37
	Eye	32	3	9.38	3.24–24.00
	Musculoskeletal	18	0	0	0.00–17.64
	**Total**	**295**	**20**	**6.78**	**4.43–10.24**
Rodents and Lagomorphs (rabbit, hare, Guinea pigs)	Skin	153	3	1.96	0.67–5.59
	Respiratory	230	5	2.17	0.94–4.93
	Urogenital	125	1	0.8	0.14–4.36
	Digestive	251	0	0	0.00–1.51
	Eye	136	1	0.74	0.13–4.09
	**Total**	**895**	**10**	**1.12**	**0.61–2.04**
Pigs	Skin	72	0	0	0.00–5.03
	Respiratory	169	1	0.59	0.10–3.25
	Urogenital	60	0	0	0.00–6.02
	Digestive	231	0	0	0.00–1.63
	Eye	42	0	0	0.00–8.31
	Musculoskeletal	18	1	5.56	0.99–25.76
	Nervous	1	1	100.0	20.65–100.00
	**Total**	**593**	**3**	**0.51**	**0.17–1.48**
Birds (poultry, water poultry, avian pets)	Skin	32	0	0	0.00–10.85
	Respiratory	86	0	0	0.00–4.25
	Urogenital	44	0	0	0.00–7.92
	Digestive	6709	12	0.18	0.10–0.31
	Eye	36	0	0	0.00–9.57
	Musculoskeletal	12	0	0	0.00–26.47
	**Total**	**6919**	**12**	**0.17**	**0.10–0.30**
Insects (bee)	Bee brood	266	2	0.75	0.21–2.70
	**Total**	**266**	**2**	**0.75**	**0.21–2.70**

**Table 2:** Prevalence of *Pantoea* spp. in all observed animals groups and organ systems in the period 2015–2017.

Animal	Organ system	Number of examined samples	Number of positive samples	Prevalence (%)	95 % CI Wilson (%)
All animals	Respiratory	1554	47	3.02	2.28–4.00
	Skin	2233	64	2.87	2.25–3.64
	Eye	740	12	1.62	0.93–2.81
	Musculoskeletal	73	1	1.37	0.24–7.36
	Bee brood	266	2	0.75	0.21–2.70
	Urogenital	743	2	0.27	0.07–0.98
	Digestive	9307	16	0.17	0.11–0.28
	Mammary gland	8822	6	0.07	0.03–0.15
	Nervous	1	1	100.0 ⁎	20.65–100.0
	**Total**	**23,739**	**151**	**0.64**	0.54–0.75

⁎ Note: The 100 % prevalence in the nervous system is based on only one sample, and thus should be interpreted with caution.

Inclusion criteria comprised samples collected from animals showing clinical symptoms within the study period and region, following standard protocols by trained personnel. Samples had to be transported under conditions preserving their integrity (at 4 °C within 24 h). Exclusion criteria included samples lacking proper documentation or collected outside established protocols; those contaminated or compromised during collection or transport; samples from asymptomatic animals or animals not part of routine examinations; and samples obtained outside the designated timeframe or geographical area.

Moreover, ethical considerations emphasized responsible sampling practices: procedures were limited to routine diagnostic needs; all personnel involved were trained in proper techniques and animal handling; informed consent was obtained from owners or caretakers; and confidentiality of animal and owner data was maintained. Efforts were made to minimize animal distress through appropriate methods, and all procedures adhered to national and institutional animal welfare regulations.

All samples were examined by cultivation methods on meat peptone blood agar (MPBA) Endo agar (EA) and xylose lysine deoxycholate agar (XLD) (all Trios Ltd. Prague, Czech Republic) and plates were incubated aerobically at 37 ± 1 °C for 24 h.

The colonies of suspected Gram-negative microorganisms were isolated on MPBA and pure microbial cultures were identified using phenotypic molecular mass spectrometry, i.e., matrix-assisted laser desorption/ionization coupled to time-of-flight mass spectrometry (MALDI-TOF MS) using a Microflex LT System spectrometer (Bruker Daltonik GmbH & Co. KG, Bremen, Germany), based on proteomics analyses, and MALDI Biotyper software MBT Compass 4.1 (Bruker Daltonik GmbH & Co. KG, Bremen, Germany).

Selected strains were tested for susceptibility to antimicrobials by disc diffusion method. The Mueller-Hinton agar (Trios Ltd. Prague, Czech Republic) and antibiotic discs were used for testing (Oxoid Ltd., Basingstoke, UK). Tested antibiotics were chloramphenicol (30 μg) tetracycline (30 μg), ampicillin (10 μg), neomycin (30 μg), gentamicin (10 μg), cefalexin (30 μg), enrofloxacin (5 μg), amoxicillin/clavulanic acid (30 μg), colostin sulphate (10 μg) and trimethoprim/sulfamethoxazole (1.25/23.75 μg per disc). Tests were assessed after 18–24 h of incubation at 37 ± 1 °C. The interpretation of values accordance to Clinical and Laboratory Standards Institute (CLSI) [22], European Committee on Antimicrobial Susceptibility Testing (EUCAST) standards [23], and literary published data [24] was performed. All used discs and mediums with reference strains *Escherichia coli* (ATCC 25922) and *Staphylococcus aureus* (ATCC 25923) were tested. The *Enterobacteriaceae* reference values are shown in Table 3.

**Table 3:** Susceptibility table - reference values for *Enterobacteriaceae.*

Antimicrobials	Antibiotics concentration per disc (μg)	Diameter (mm)		
		R	S	Source
Amoxicillin/clavulanic acid (*Enterobacteriaceae*)	20/10	≤13	≥18	CLSI VET 01 S (2018)
Ampicillin (*Enterobacteriaceae*)	10	≤13	≥17	CLSI VET 01 S (2018)
Cefalexin (*Enterobacteriaceae*)	30	<14	≥14	EUCAST (2022)
Neomycin (*Enterobacteriaceae*)	30	≤15	≥17	CASFM VET (2018)
Gentamicin (*Enterobacteriaceae*)	10	≤12	≥16	CLSI VET 01 S (2018)
Chloramphenicol (*Enterobacteriaceae*)	30	≤12	≥18	CLSI VET 01 S (2018)
Tetracycline (*Enterobacteriaceae*)	30	≤11	≥15	CLSI VET 01 S (2018)
Enrofloxacin (*Enterobacteriaceae*)	5	≤16	≥23	CLSI VET 01 S (2018)
Colistin (*Enterobacteriaceae*)	10	≤11	≥14	Galani et al. (2008)
*Trimethoprim/sulfamethoxazole (*Enterobacteriaceae*)	1.25/23.75	≤10	≥16	CLSI VET 01 S (2018)

S=Susceptible; R = Resistant; ^⁎^ Co-trimoxazole.

Descriptive statistics were used to calculate the prevalence of *Pantoea* spp. across animal species and organ systems. Prevalence was expressed as the percentage of positive samples out of the total number examined. To assess whether differences in prevalence among species and organ systems were statistically significant, Pearson's chi-square (χ^2^) test was performed. A *p*-value of <0.05 was considered statistically significant. In addition, 95 % confidence intervals (CI) for prevalence estimates were calculated using the Wilson score method to account for variability, particularly in groups with small sample sizes. All statistical analyses were conducted using software IBM SPSS Statistics version 30.

## 3 Results

The study of the prevalence and fenotypic resistance characterization of *Pantoea* strains in animal populations provides critical insights into the complex dynamics of this pathogen, revealing its potential impact on the animals, environment and human health. From a total of 23,739 samples of clinical veterinary material obtained in the years 2015–2017, 151 strains of *Pantoea* spp., a probable species of *Pantoea agglomerans* (prevalence 0.63 %), were isolated from these materials. The MALDI TOF MS value score for the detected strains ranged between 2.003 and 2.259 (all secure genus identification and probable species identification). For 18 strains, pure cultures of the causative agent grown in strong intensity were recorded in the examined materials, of which in 16 cases a solo occurrence was recorded in dogs, in 1 case in ruminants and in 1 case in an ornamental bird. In the remaining cases, *Pantoea* spp. strains were present in a mixture with 1 to 4 other types of microorganisms.

From domestic carnivores, 4643 clinical samples were examined and a total of 76 strains (prevalence 1.64 %) of *Pantoea* spp. Most strains (n = 71) were isolated from dogs (*Canis familiaris*). In dogs, the most frequent detections were in skin swabs, ear swabs and nasal swabs (*n* = 37, 11 and 7 respectively). Isolations from eye swabs, rectal swabs, bronchial lavages, claw swabs and skin abscesses were less common (*n* = 7, 3, 2, 2 and 2 respectively). 5 strains were isolated from cats (*Felis catus*), 2 from a nasal swab, 2 from a skin swab and 1 from an eye. Pathological lesions were the most diverse of all animals in carnivores. Among the respiratory symptoms, rhinitis, purulent rhinitis, chronic rhinitis, haemorrhagic rhinitis, long-term cough, sinusitis, chronic nasal discharge were recorded, the eyes showed symptoms of acute conjunctivitis, the symptoms of skin diseases were very rich, manifested by purulent, alopecia, squamous, recurrent dermatitis, by the formation of abscesses, deep pyodermas, non-healing lesions, chronic and recurrent otitis media, or exungulation of claws. Repeated watery diarrhoea has been described when the digestive system is affected.

Regarding domestic ruminants, 10,128 clinical samples were examined in the monitored period, from which a total of 28 strains of *Pantoea* spp. (prevalence 0.28 %), of which 20 from cattle (*Bos taurus*) and 8 from goats (*Capra hircus*). In the case of cattle, 6 isolates came from nasal swabs and 2 isolates from tracheal swab and 2 from bronchioalveolar lavage of calves, 5 isolates from milk, 3 from nasal swab, and 1 from bronchial lavage of dairy cows. One isolate came from the nasal swab of a bull. Of the 8 strains isolated from goats, 4 isolates came from nasal swab, 2 from skin, 1 from pharyngeal swab and 1 from milk. Symptoms of acute mastitis, difficulty breathing, cough, rhinitis, pneumonia and other unspecified respiratory diseases were recorded in the cattle. In goats, rhinitis, cough, hoarseness, dermatitis and acute mastitis.

The domestic equids represented by horses (*Equus caballus f. caballus*), 295 clinical samples were examined, from which a total of 20 strains of *Pantoea* spp. (prevalence 6.78 %), of which 9 isolates came from nasal swabs, 2 from sputum, 3 from eye swabs, 5 isolates from skin swabs and 1 from urine. Respiratory problems, cough, recurrent discharge from the nostrils, rhinitis chronica, bronchopneumonia, conjunctivitis, dermatitis and dermatitis chronica and cystitis were recorded as pathological processes in horses. This is relevant because the involvement of *P. agglomerans* that has previously been documented as a cause of bacterial placentitis, but recently it has been evidenced as a cause of abortion in horses [25].

From domestic rodents and hares (rabbit, guinea pig, hare), 895 clinical samples were examined and a total of 10 strains of *Pantoea* spp. (prevalence 1.12 %) of which 1 strain from a hare (*Lepus europaeus*) testicle smear, 1 strain from a guinea pig (*Cavia aperea f. porcellus*) skin smear. A total of 8 strains were isolated from rabbits (*Oryctolagus cuniculus* f. domestica), of which 5 strains came from nasal swabs, 1 strain from the eye and 2 strains from ear swabs. Purulent orchitis was noted in a hare. In rabbits, acute rhinitis was diagnosed in 3 cases, rhinitis chronica in 1 case, bronchitis chronica in 1 case, otitis in 2 cases and swelling of the eyes and nostrils in 1 case. The guinea pig showed symptoms of apruritic dermatitis.

A total of 593 clinical samples from pigs (*Sus scrofa f. domestica*) were examined in the monitored period and 3 strains of *Pantoea* spp. (prevalence 0.51 %). One strain came from a nasal swab, one from a joint punctate, and one from cerebrospinal fluid. Symptoms of bronchopneumonia, arthritis and meningitis have been reported.

From birds (waterfowl, ratites, ornamental birds), 6919 clinical samples were examined and 12 strains of *Pantoea* spp. (prevalence 0.17 %). One isolate from budgerigar (*Melopsittacus undulatus*) organ smears, one isolate from liver smear from pigeon (*Columba livia f. domestica*), two isolates from liver smear from hen *(Gallus gallus f. domestica*), one isolate from cloacal swab of a shrike (*Urocissa erythrorhyncha*), one from bearded dragon droppings (*Lybius dubius*), one from muskrat droppings (*Cairina moschata f. domestica*), one from nestling droppings (*Nestor notabilis*), one from goose droppings (*Alopochen aegyptiacus*), one from neophema droppings (*Neophema pulchella*) and two from cloacal swabs and droppings of an unspecified parrot species. Diarrheal diseases accompanied by cachexia or anaemia were diagnosed in all cases in the above-mentioned birds.

In the case of insects, 266 bee brood samples were examined in the monitored period and 2 strains of *Pantoea* spp. were isolated. (prevalence 0.75 %), from two bee colonies (*Apis mellifera)* in which brood death was recorded and American foulbrood was suspected.

Table 1, Table 2 show a precise overview of the numbers of examined samples and prevalence according to individual groups of animals and organs. Of the 18 strains grown in pure cultures, 16 strains were isolated at high intensity from dogs, 1 from a calf and 1 from a parrot. A total of 8 of them were isolated from skin lesions, of which seven cases were diagnosed with acute dermatitis, one case with squamous dermatitis and one case with pustular dermatitis. In 4 cases, strains were isolated from the external auditory canal and symptoms of otitis externa acute were described in two cases, otitis externa chronica in one case, and otitis externa sicca in one case. In 3 cases, the strains came from eye smears, of which conjunctivitis acuta was diagnosed in two cases, and corneal opacity with the formation of corneal erosions in one case. In 1 case, the isolate was obtained from a nasal swab with a diagnosis of rhinitis purulent. In 1 case, a pure culture was isolated from the nasal swab of a calf with symptoms of acute bronchopneumonia and in 1 case from the droppings of a *Nestor notabilis* parrot with symptoms of watery diarrhoea.

**Fig. 1:**
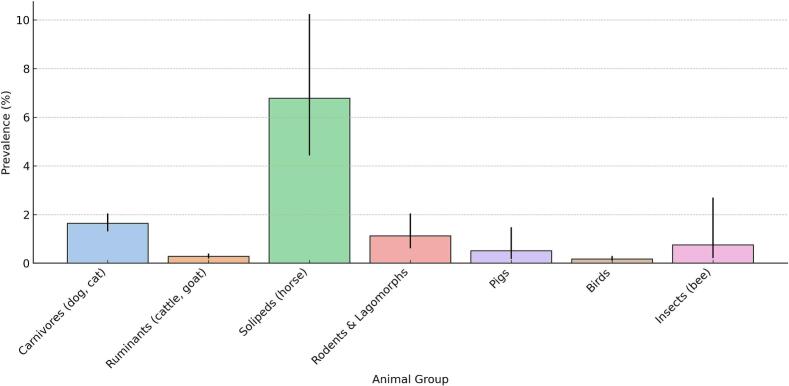
Prevalence of *Pantoea agglomerans* by animal group (2015–2017).

To assess the distribution of *Pantoea* spp. across different animal hosts and organ systems, chi-square tests and 95 % confidence intervals (CIs) were used to evaluate statistical significance and estimate prevalence reliability. The prevalence of *Pantoea* spp. differed significantly among animal species (χ^2^ = 297.39, df = 6, *p* < 0.001). The highest prevalence was observed in horses (6.78 %; 95 % CI: 4.43–10.24 %), followed by carnivores (1.64 %; 95 % CI: 1.31–2.04 %) and rodents/lagomorphs (1.12 %; 95 % CI: 0.61–2.04 %). The lowest rates were recorded in birds (0.17 %; 95 % CI: 0.10–0.30 %), ruminants (0.28 %; 95 % CI: 0.19–0.40 %), and pigs (0.51 %; 95 % CI: 0.17–1.48 %). The results are shown in Fig. 1. These results confirm non-random species-specific variation in *Pantoea* spp. detection.

Significant variation was also observed across organ systems (χ^2^ = 562.56, df = 8, *p* < 0.001). The respiratory tract (3.02 %; 95 % CI: 2.28–4.00 %) and skin (2.87 %; 95 % CI: 2.25–3.64 %) had the highest prevalence. Other affected systems included the eye (1.62 %; 95 % CI: 0.93–2.81 %), musculoskeletal system (1.37 %; 95 % CI: 0.24–7.36 %), and bee brood (0.75 %; 95 % CI: 0.21–2.70 %). The digestive (0.17 %; 95 % CI: 0.11–0.28 %), urogenital (0.27 %; 95 % CI: 0.07–0.98 %), and mammary gland systems (0.07 %; 95 % CI: 0.03–0.15 %) showed the lowest prevalence. While the nervous system exhibited a 100 % positivity rate, this was based on a single sample and should be interpreted cautiously.

Together, these results highlight distinct patterns in host and tissue susceptibility to *Pantoea* spp., reinforcing the importance of targeted surveillance and the potential for species- and organ-specific pathogenicity in veterinary contexts.

**Fig. 2:**
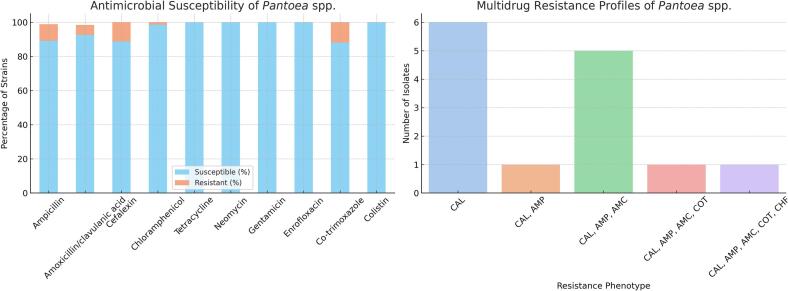
Antimicrobial susceptibility and multidrug resistance profiles of *Pantoea* spp. strains. Legend: CAL – cefalexin, AMP – ampicilin, AMC - amoxicillin/clavulanic acid, COT - sulfamethoxazole/trimethoprim; CHF – chloramphenicol.

The best sensitivities of isolated strains of *Pantoea* spp. were detected to tetracycline, gentamicin, enrofloxacin, neomycin and colistin (100 % sensitive strains), antimicrobial resistance was recorded in 14 tested strains, namely in the case of chloramphenicol, amoxicillin/clavulanic acid, ampicillin, cephalexin and co-trimoxazole (resistance 1.4, 7.3, 10.8, 11.2 and 11.8 % of tested strains respectively). Table 4 shows the summary results. In 12 cases, resistance was detected only within the group of beta-lactam antibiotics. Resistance to 2 or more groups of antimicrobial agents was evaluated as multiresistance and was detected in two isolated strains of *Pantoea* spp. The results are shown in Fig. 2. In the first multiresistant strain, resistance to 4 and in the second case to 5 tested antimicrobial substances was detected (see Table 5). Repeated watery diarrhoea was described in one of the multiresistant strains isolated from a rectal swab of a dog. This strain showed resistance to ampicillin, amoxicillin with clavulanic acid, cephalexin and co-trimoxazole. At the same time, *E. coli* was also isolated in this case in a weak intensity. In the second multiresistant strain of *Pantoea* spp. isolated from the skin of a dog, the formation of scales and pustules on the skin was detected. In this case, multi-resistance to ampicillin, amoxicillin with clavulanic acid, cephalexin, co-trimoxazole and chloramphenicol was noted, and it was a strain that also grew in pure culture.

**Table 4:** Susceptibility of *Pantoea* spp. strains isolated from animals in period 2015–2017.

Antimicrobials	Number of examined strains	Susceptible	Intermediary	Resistant
Ampicillin	83	74 (89.2 %)	1 (1.2 %)	8 (9.6 %)
Amoxicillin/clavulanic acid	124	115 (92.7 %)	2 (1.6 %)	7 (5.7 %)
Cefalexin	125	111 (88.8 %)	0	14 (11.2 %)
Chloramphenicol	71	70 (98.6 %)	0	1 (1.4 %)
Tetracycline	129	129 (100 %)	0	0
Neomycin	99	99 (100 %)	0	0
Gentamicin	128	128 (100 %)	0	0
Enrofloxacin	124	124 (100 %)	0	0
⁎Trimethoprim/sulfamethoxazole	17	15 (88.2 %)	0	2 (11.8 %)
Colistin	37	37 (100 %)	0	0

⁎ Co-trimoxazole.

**Table 5:** Resistance profile of *Pantoea* spp. isolated strains.

Frequency of resistance by		Phenotype of resistance	Number of isolates
Active substance	Antimicrobial group		
1	1	CAL	6
2	1	CAL, AMP	1
3	1	CAL, AMP, AMC	5
4	2	CAL, AMP, AMC, COT	1*
5	3	CAL, AMP, AMC, COT, CHF	1*
Sum of multidrug – resistant strains			2*

Resistance profiles for *Pantoea* species clinical isolates (*n* = 14);

AMP - ampicilin, AMC - amoxicillin/clavulanic acid, CAL - cefalexin, CHF – chloramphenicol, COT - sulfamethoxazole/trimethoprim; *Multidrug resistant isolates, i.e. resistance to ≥2 groups of antimicrobials.

## 4 Discussion

The observed differences in *Pantoea* spp. prevalence across animal species and organ systems were statistically significant, suggesting non-random patterns of occurrence. The highest prevalence of *Pantoea* spp. was observed in horses (6.78 %), domestic carnivores (1.64 %), and domestic rodents, including hares (1.12 %). The high prevalence found in carnivores is unexpected, as these animals typically have limited contact with plant material compared to herbivores like ruminants, pigs, and birds. This is noteworthy given that *Pantoea* spp. infections in humans are frequently linked to exposure to plant material, particularly following penetrating trauma [14]. In contrast, lower prevalence rates were found in domestic birds (0.17 %), ruminants (0.28 %), and pigs (0.51 %). These findings point toward the possibility of alternative transmission routes or reservoirs in veterinary environments and supports the idea that host-specific factors, including immune status, environment, and management practices, may influence susceptibility to *Pantoea* colonization or infection.

In terms of anatomical distribution, *Pantoea* spp. were most commonly found in cerebrospinal fluid samples from the nervous system (100 %), respiratory tract (3.02 %) and skin (2.87 %). However, the 100 % prevalence in the nervous system is based on a single cerebrospinal fluid sample from a pig, limiting the reliability of this observation. Nevertheless, the findings of higher prevalence in the respiratory tract and skin lesions are consistent with previously published reports of *Pantoea* spp. involvement in respiratory and dermatological conditions in animals and humans [15,16]. Interestingly, human scientific publications often mention the capture of *Pantoea* spp. from blood cultures during bacteraemia's [[12], [13], [14]]. On the contrary, the lowest prevalence was recorded in our case in samples from the mammary gland (0.07 %), digestive tract (0.17 %), and urogenital tract (0.27 %). These findings support the notion that *Pantoea* spp., though traditionally overlooked in veterinary contexts, can be isolated from a broad spectrum of animal hosts and organs. These statistically validated trends emphasize the relevance of *Pantoea* spp. in clinical veterinary diagnostics and suggest a broader ecological niche than previously assumed. This aligns with earlier reports of *P. agglomerans* causing equine placentitis [17] and haemorrhagic disease in marine fish [18], suggesting that members of this genus may act as opportunistic animal pathogens.

Our antimicrobial susceptibility data are largely consistent with previously published findings [14,20]. In contrast, some human strains of *Pantoea* showed more extensive forms of multiresistance to up to 4 groups of antimicrobials, such as penicillins, aminoglycosides, fluoroquinolones and even carbapenems [15]. Even though *Pantoea agglomerans* is a low-virulence pathogen, even in an immunocompromised adult host, it causes a diverse clinical picture and can be successfully treated with the proper use of antibiotics [26]. Nevertheless, the emergence of resistant strains underscores the potential public health implications, particularly in the context of zoonotic transmission [27,28].

Although *Pantoea* spp. are rarely reported in association with animal diseases, our study contributes novel insights by documenting their occurrence in diverse animal hosts and organ systems. Notably, over 10 % of isolates were obtained as pure cultures from clinically significant lesions, reinforcing the potential pathogenic role of these organisms in animals.

The present study has several limitations that should be acknowledged. The reliance on culture-based methods and MALDI-TOF MS without molecular species confirmation may lead to potential misidentification, particularly in cases involving closely related species or mixed infections. Additionally, the observational design precludes the establishment of causal relationships and may introduce sampling bias, as specimens were collected exclusively from animals exhibiting clinical signs in specific regions of the Czech Republic. As a result, the findings may not be fully generalizable to other geographic areas or to asymptomatic populations. Although *Pantoea* genus bacteria have been isolated from infections in both animals and humans, the potential for transmission between species remains unclear, and any interpretations in this regard should be made with the utmost caution. Furthermore, the detection of *Pantoea* spp. in clinical samples does not confirm it as the primary cause of the observed symptoms. The absence of thorough etiological investigations limits the ability to accurately determine causality.

Despite these limitations, the study has several notable strengths. It provides valuable baseline data on the prevalence and diversity of *Pantoea* spp. pathogens in clinically affected animals in Czech Republic, contributing important knowledge to the field of veterinary infectious disease epidemiology. The use of MALDI-TOF MS enabled rapid and cost-effective identification of a wide range of pathogens, demonstrating its practical utility in routine diagnostic settings. In addition, the focus of the study on naturally occurring clinical cases adds ecological relevance and enhances the applicability of the findings to real veterinary practice. Importantly, the identification of potentially zoonotic pathogens highlights the need for integrated surveillance approaches, as the findings may have public health implications through the possible transmission of infectious agents from animals to humans, particularly in agricultural or rural settings. Future research using molecular diagnostics and broader sampling would help to address these limitations and provide a more comprehensive understanding of pathogen distribution.

## 5 Conclusion

The implementation of integrated surveillance systems that include veterinary, clinical and environmental data on *Pantoea* spp. strains is essential not only to infection control in animals but also to mitigate risks to human populations, highlighting the need for interdisciplinary collaboration to address antimicrobial resistance and ensure public health safety. More detailed work is needed that also maps the genotypic make-up of veterinary origin and the associated ability to produce virulence factors, antimicrobial resistance and other physiological capabilities that influence or are responsible for pathogenicity in animal patients.

## Originality and prior publication

This manuscript is original and has not been published previously nor is it currently under consideration for publication elsewhere, in whole or in part.

## CRediT authorship contribution statement

**Ondrej Holy:** Writing – original draft, Methodology, Funding acquisition, Formal analysis. **Vladimir Sladecek:** Writing – original draft, Methodology, Formal analysis. **Jaroslav Bzdil:** Writing – original draft, Methodology, Investigation, Data curation. **Monika Zouharova:** Methodology, Formal analysis, Data curation. **Julio Parra-Flores:** Writing – original draft, Methodology. **Cátia Caneiras:** Writing – original draft, Methodology, Formal analysis, Data curation.

## Consent for publication

All authors consent to the publication of this manuscript in *One Health*.

## Ethics

The study followed all relevant institutional and national guidelines.

## Authorship

All authors have made substantial contributions to the conception, design, data acquisition, analysis, or interpretation of the work; have participated in drafting or revising the manuscript; and have approved the final version for submission.

## Funding

The work was supported by the project ‘Interdisciplinary Approaches to the Prevention and Diagnosis of Viral Diseases’ (CZ.02.01.01/00/23_021/0008856), funded by the 10.13039/501100008530European Regional Development Fund (ERDF) under the Johannes Amos Comenius Programme. We also acknowledge support from 10.13039/501100008530ERDF/ESF Project TECHSCALE (Grant CZ.02.01.01/00/22_008/0004587).

## Declaration of competing interest

The authors declare that they have no known competing financial interests or personal relationships that could have appeared to influence the work reported in this paper.

## Data Availability

The data supporting the findings of this study are available within the article. Further details may be provided upon reasonable request to the corresponding author.
